# Can Artificial Intelligence Be Applied to Diagnose Intracerebral Hemorrhage under the Background of the Fourth Industrial Revolution? A Novel Systemic Review and Meta-Analysis

**DOI:** 10.1155/2022/9430097

**Published:** 2022-02-24

**Authors:** Kai Zhao, Qing Zhao, Ping Zhou, Bin Liu, Qiang Zhang, Mingfei Yang

**Affiliations:** ^1^Graduate School, Qinghai University, Xining 810016, Qinghai, China; ^2^Human Resource, Women's and Children's Hospital of Qinghai Province, Xining 810007, Qinghai, China; ^3^Department of Neurosurgery, Qinghai Provincial People's Hospital, Xining 810007, Qinghai, China

## Abstract

**Aim:**

We intended to provide the clinical evidence that artificial intelligence (AI) could be used to assist doctors in the diagnosis of intracerebral hemorrhage (ICH).

**Methods:**

Studies published in 2021 were identified after the literature search of PubMed, Embase, and Cochrane. Quality Assessment of Diagnostic Accuracy Studies-2 (QUADAS-2) was used to perform the quality assessment of studies. Data extraction of diagnosis effect included accuracy (ACC), sensitivity (SEN), specificity (SPE), positive predictive value (PPV), negative predictive value (NPV), area under curve (AUC), and Dice scores (Dices). The pooled effect with its 95% confidence interval (95%CI) was calculated by the random effects model. *I*-Square (*I*^2^) was used to test heterogeneity. To check the stability of the overall results, sensitivity analysis was conducted by recalculating the pooled effect of the remaining studies after omitting the study with the highest quality or the random effects model was switched to the fixed effects model. Funnel plot was used to evaluate publication bias. To reduce heterogeneity, recalculating the pooled effect of the remaining studies after omitting the study with the lowest quality or perform subgroup analysis.

**Results:**

Twenty-five diagnostic tests of ICH via AI and doctors with overall high quality were included. Pooled ACC, SEN, SPE, PPV, NPV, AUC, and Dices were 0.88 (0.83∼0.93), 0.85 (0.81∼0.89), 0.90 (0.88∼0.92), 0.80 (0.75∼0.85), 0.93 (0.91∼0.95), 0.84 (0.80∼0.89), and 0.90 (0.85∼0.95), respectively. There was no publication bias. All of results were stable as revealed by sensitivity analysis and were accordant as outcomes via subgroups analysis.

**Conclusion:**

Under the background of the fourth industrial revolution, AI might be an effective and efficient tool to assist doctors in the clinical diagnosis of ICH.

## 1. Introduction

Appearance of the fourth industrial revolution was based on the digitization and big data analysis [1]. The typical representatives were artificial intelligence (AI) and blockchain [2]. Without exception, there were more and more AI technologies or various software applied in medicine, especially in medical imageology [3]. Stroke was a major cause of death and disability globally; in particular, hemorrhagic strokes (including intracerebral and subarachnoid hemorrhage) had a relatively stable incidence adjusted for age in high-income countries but an increasing incidence in low-income and middle-income countries each year [4]. Of the 15 million strokes reported worldwide annually, intracerebral hemorrhage (ICH) accounts for approximately 10% to 15% of all stroke cases in the United Statement, Europe, and Australia and approximately 20% to 30% of strokes in Asia [5]. The median 30-day mortality rate after ICH is approximately 15–50%, and only 20% of patients regain functional independence within three months after the ictus [6]. Therefore, ICH, as a stroke subtype with high mortality and poor functional outcome in survivors, needed the accurate and objective evidence of neuroimaging to make a definite diagnosis [7]. AI used to diagnose ICH based on neuroimaging gradually became a trend to promote the development of intelligent medicine and efficiency of clinicians recently [8]. Apart from economic interest and development of AI industries, in the aspect of diagnostics, there was no evidence that AI could assist doctors in practically clinical work. In view of that the development of AI industries was quick as a flash, we intend to perform a novel systemic review and meta-analysis based on recent diagnostic tests, which were able to represent the state of the art AI technologies, to verify the hypothesis that AI might be an effective and efficient tool to diagnose ICH.

## 2. Materials and Methods

### 2.1. Search Strategy

Literature search was performed in three public electronic databases of PubMed, Embase and Cochrane. The strategy of literature search was as follows: (((((((((((((((((Intelligence, Artificial[Title/Abstract]) OR (“Artificial Intelligence”[Mesh])) OR (Computational Intelligence[Title/Abstract])) OR (Intelligence, Computational[Title/Abstract])) OR (Machine Intelligence[Title/Abstract])) OR (Intelligence, Machine[Title/Abstract])) OR (Computer Reasoning[Title/Abstract])) OR (Reasoning, Computer[Title/Abstract])) OR (AI (Artificial Intelligence)[Title/Abstract])) OR (Computer Vision System^*∗*^[Title/Abstract])) OR (System^*∗*^, Computer Vision[Title/Abstract])) OR (Vision System^*∗*^, Computer[Title/Abstract])) OR (Knowledge Acquisition (Computer)[Title/Abstract])) OR (Acquisition, Knowledge (Computer)[Title/Abstract])) OR (Knowledge Representation^*∗*^ (Computer)[Title/Abstract])) OR (Representation, Knowledge (Computer)[Title/Abstract])) OR ((((“Machine Learning”[Mesh]) OR (Learning, Machine[Title/Abstract])) OR (Transfer Learning[Title/Abstract])) OR (Learning, Transfer[Title/Abstract]))) AND (((((((((((((“Cerebral Hemorrhage”[Mesh]) OR (Hemorrhage^*∗*^, Cerebrum[Title/Abstract])) OR (Cerebrum Hemorrhage^*∗*^[Title/Abstract])) OR (Cerebral Parenchymal Hemorrhage^*∗*^[Title/Abstract])) OR (Hemorrhage^*∗*^, Cerebral Parenchymal[Title/Abstract])) OR (Parenchymal Hemorrhage^*∗*^, Cerebral[Title/Abstract])) OR (Intracerebral Hemorrhage^*∗*^[Title/Abstract])) OR (Hemorrhage^*∗*^, Intracerebral[Title/Abstract])) OR (Hemorrhage^*∗*^, Cerebral[Title/Abstract])) OR (Cerebral Hemorrhages[Title/Abstract])) OR (Brain Hemorrhage^*∗*^, Cerebral[Title/Abstract])) OR (Cerebral Brain Hemorrhage^*∗*^[Title/Abstract])) OR (Hemorrhage^*∗*^, Cerebral Brain[Title/Abstract])).

### 2.2. Inclusion Criteria

(1) Language and regions of articles were not restricted; (2) articles were published in 2021; (3) diagnostic tests; (4) true-positive participates were patients suffered ICH; (5) true-negative participates were people without abnormal condition in neuroimaging; (6) the gold standard was that professional physicians, who were blind to tests, diagnose ICH or no ICH referring to the International Classification of Diseases and recent international standards guidelines; (7) full-automatic or semi-automatic diagnostic conclusions via AI technologies were used to compare with full-manual diagnostic outcomes via professional physician; (8) analysis or assessment of diagnosis effect was performed completely.

### 2.3. Exclusion Criteria

(1) Duplication; (2) reviews, comments, letters, case reports, protocols of clinic trials, and conference papers; (3) animal experiments; (4) and contents of articles were irrelevant to this meta-analysis.

### 2.4. Quality Assessment

The quality assessment of the included articles was performed via the Quality Assessment of Diagnostic Accuracy Studies-2 (QUADAS-2) by the software Review Manager 5.3 before data extraction. We considered that the study might be assessed to have higher quality for its larger number of included patients in studies with the same assessment in QUADAS-2.

### 2.5. Data Extraction

All the original data used to assess diagnosis effect were extracted including accuracy (ACC), sensitivity(SEN), specificity (SPE), positive predictive value (PPV), negative predictive value (NPV), area under curve (AUC), and Dice scores (Dices),. In addition, some confounders, which might result in errors, were adjusted, including different diagnosis purposes, AI technologies, and other factors.

### 2.6. Statistical Analysis

Relative numbers and their 95% confidence intervals (95%CI) were used to describe count data. Meta-analysis was performed using corresponding modules in Software for Statistics and Data Science (Stata, version 15.1; College Station, Texas 77845 USA). The pooled effect with its 95%CI was calculated by the random effects model. *I*-Square (*I*^2^) was used to test the heterogeneity. Sensitivity analysis was performed to evaluate the stability of overall results by recalculating the pooled effect of the remaining studies after omitting the study with the highest quality or the random effects model was switched to fixed effects model. Funnel plot symmetry and Egger's regression were used to evaluate publication bias. To reduce heterogeneity, recalculating the pooled effect of the remaining studies after omitting the study with the lowest quality or perform subgroups analysis. All *p* values were two-sided with a significant level at 0.05.

## 3. Results

### 3.1. Literature Search and Study Characteristics

**Figure 1:**
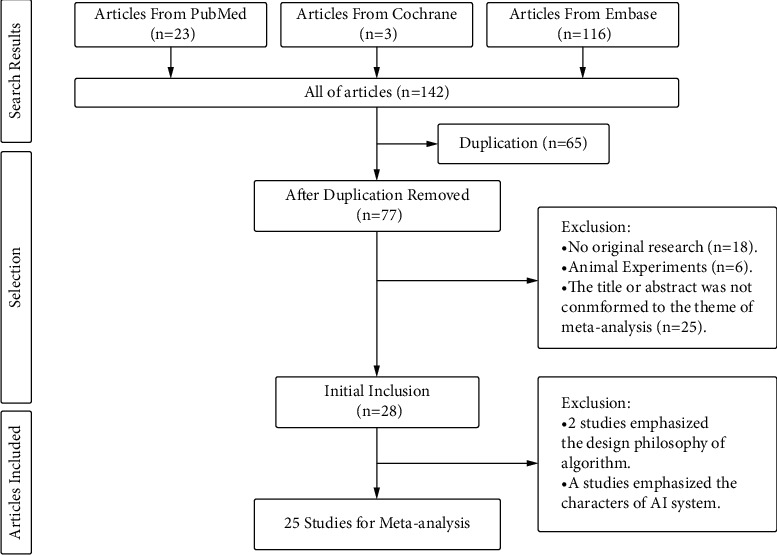
Process of literature search.

Totally, 142 articles were retrieved from 3 databases according to the strategy. After screening according to the inclusion and exclusion criteria, 25 articles [9–33] of diagnostic tests were enrolled ultimately (Figure 1). A total of 23071 ICH patients participated in all the tests, who were manually diagnosed by professional physicians referring to the gold standard of ICH diagnosis in the latest international clinical guidelines (Table 1). 24 AI technologies or methods based on clinical features and neuroimaging were participate in all the tests. The aims of the tests were classified into 4 main aspects: detection of ICH, segmentation of ICH in neuroimaging, prediction of prognosis, and hematoma enlargement in ICH patients. The conclusion with the same tendency was that AI could effectively assist diagnosis of ICH. Specially, four articles (Lu Li, Yu Lei, Stefan Pszczolkowski, Masahito Katsuki) included two independent data extraction. Lu Li's study separated hematoma volume to “big” and “small” groups to study independently. Yu Lei's study studied the risk of ICH and occurrence of ICH independently. Stefan Pszczolkowski' study had two study aims independently: detection of ICH and prediction of prognosis in ICH patients. Masahito Katsuki wrote 2 different articles as the same first author.

### 3.2. Quality Assessment of Studies

**Figure 2:**
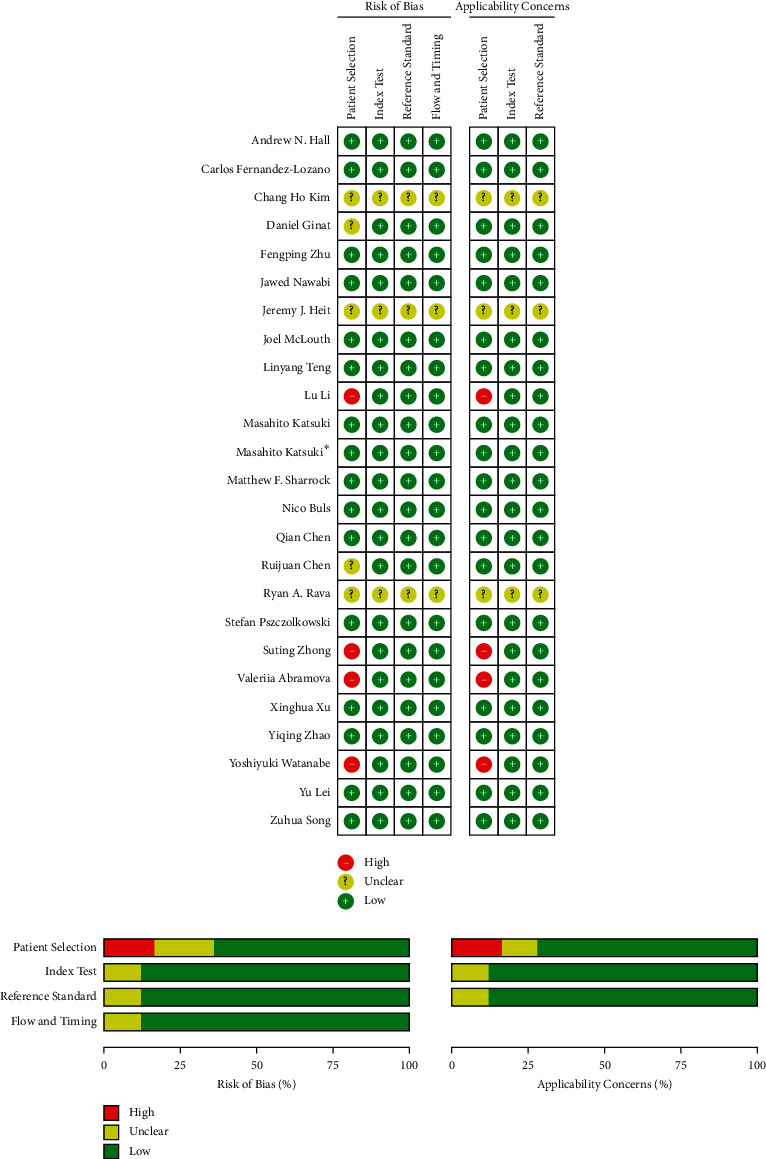
Quality assessment of studies via quality assessment of diagnostic accuracy studies-2.

The assessment of article quality via QUADAS-2 is shown in Figure 2. In the Risk of Bias section, four studies (Lu Li, Suting Zhong, Valeriia Abramova, Yoshiyuki Watanabe) were evaluated as high risk and five studies (Chang Ho Kim, Jeremy J. Heit, Ryan A. Rava, Ruijuan Chen, Daniel Ginat) were evaluated as unclear risk in the Patient Selection segment, and in addition, three studies (Chang Ho Kim, Jeremy J. Heit, Ryan A. Rava) were assessed to unclear risk in other segments. In the Applicability Concerns section, four studies (Lu Li, Suting Zhong, Valeriia Abramova, Yoshiyuki Watanabe) were evaluated as high concern and three studies (Chang Ho Kim, Jeremy J. Heit, Ryan A. Rava) were evaluated as unclear concern in the Patient Selection segment, and in addition, three studies (Chang Ho Kim, Jeremy J. Heit, Ryan A. Rava) were assessed to unclear risk in other segments. Except outcomes of the assessment above, any segment was assessed to low risks in the Risk of Bias section or low concerns in the Applicability Concerns section as well as other studies.

### 3.3. Data Analysis

**Figure 3:**
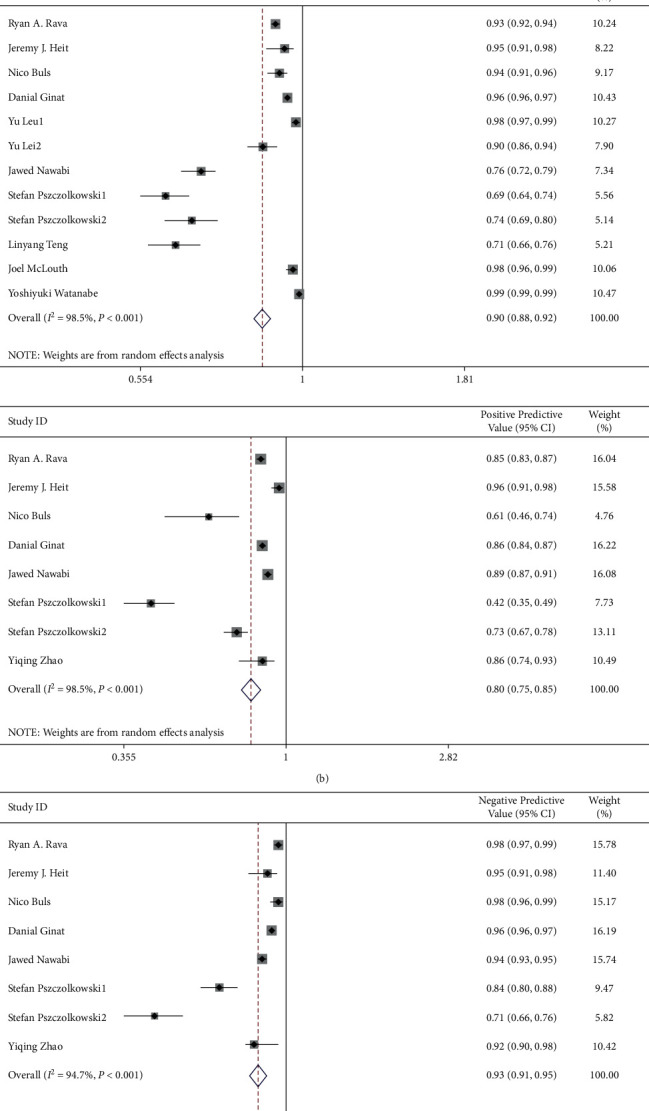
Pooled accuracy/sensitivity/specificity/positive predictive value/negative predictive value/area under curve/dice scores of artificial intelligence used in ICH diagnosis.

Total pooled ACC, SEN, SPE, PPV, NPV, AUC, and Dices were 0.88 (0.83∼0.93), 0.85 (0.81∼0.89), 0.90 (0.88∼0.92), 0.80 (0.75∼0.85), 0.93 (0.91∼0.95), 0.84 (0.80∼0.89), and 0.90 (0.85∼0.95). Heterogeneity of pooled ACC, SEN, SPE, PPV, NPV, AUC, and Dices were 98.6% (*p* < 0.001), 95.9% (*p* < 0.001), 98.5% (*p* < 0.001), 95.1% (*p* < 0.001), 94.7% (*p* < 0.001), 98.1% (*p* < 0.001), and 28.5% (*p*=0.237), respectively (Figure 3).

### 3.4. Publication Bias and Sensibility Analysis

**Figure 4:**
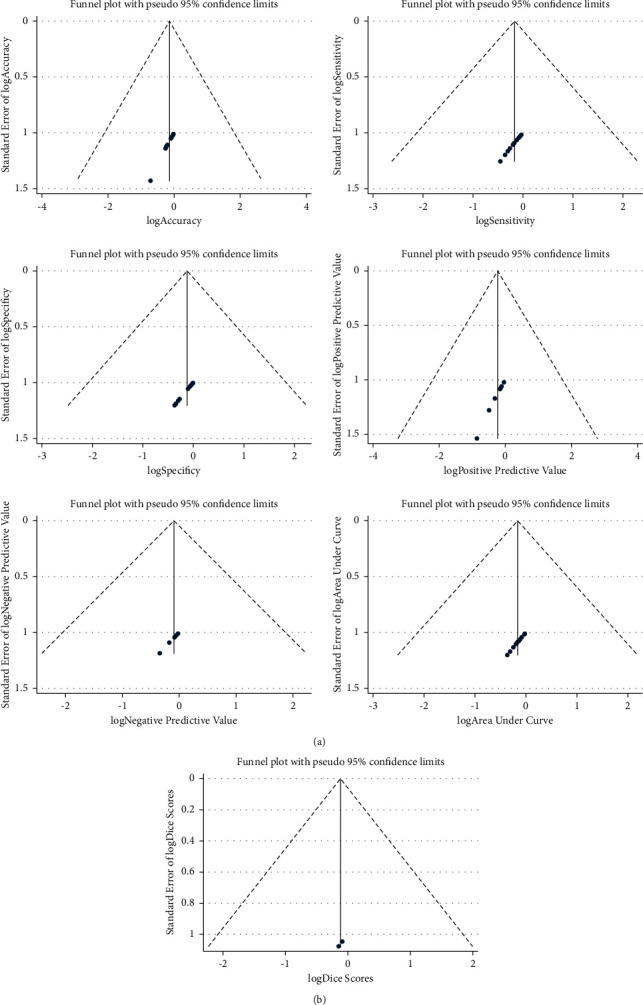
Funnel plots of overall accuracy/sensitivity/specificity/positive predictive value/negative predictive value/area under curve/dice scores of artificial intelligence used in ICH diagnosis.

There was symmetrical distribution in funnel plots (Figure 4). In sensibility analysis, after the study with the highest quality omitted or random effect model was transformed to the fixed effect model, pooled ACC (Fengping Zhu), SEN (Linyang Teng), SPE (Linyang Teng), PPV (Stefan Pszczolkowski), NPV (Stefan Pszczolkowski), AUC(Linyang Teng), and Dices (no article omitted because only 2 articles were included to perform meta-analysis of Dices) were 0.87 (0.82∼0.92) or 0.92 (0.92∼0.93), 0.85 (0.81∼0.90) or 0.88 (0.87∼0.89), 0.91 (0.89∼0.93) or 0.99 (0.99∼0.99), 0.88 (0.84∼0.91) or 0.87 (0.86∼0.88), 0.96 (0.95∼0.97) or 0.96 (0.96∼0.97), 0.85 (0.80∼0.89) or 0.89 (0.89∼0.90), and 0.90 (0.87∼0.94). Heterogeneity of pooled ACC, SEN, SPE, PPV, NPV, AUC, and Dices in sensibility analysis was 98.7% (*p* < 0.001) or 98.6% (*p* < 0.001), 96.0% (*p* < 0.001) or 95.9% (*p* < 0.001), 98.5% (*p* < 0.001) or 98.5% (*p* < 0.001), 88.9% (*p* < 0.001) or 95.1% (*p* < 0.001), 87.8% (*p* < 0.001) or 94.7% (*p* < 0.001), 97.8% (*p* < 0.001) or 98.1% (*p* < 0.001), and 28.5% (*p*=0.235) (Table 2).

### 3.5. Subgroups Analysis

Due to high heterogeneity companying, the study with the lowest quality might be the source of this phenomenon. After those studies omitted in the meta-analysis of ACC (Yoshiyuki Watanabe), SEN (Yoshiyuki Watanabe), SPE (Yoshiyuki Watanabe), PPV (Ryan A. Rava), NPV (Ryan A. Rava), and AUC (Zuhua Song), pooled effects were 0.88 (0.83∼0.94), 0.86 (0.81∼0.90), 0.88 (0.88∼0.91), 0.78 (0.72∼0.84), 0.92 (0.89∼0.94), and 0.84 (0.79∼0.89) with the heterogeneity of 98.7% (*p* < 0.001), 96.2% (*p* < 0.001), 97.4% (*p* < 0.001), 95.7% (*p* < 0.001), 94.8% (*p* < 0.001), and 98.2% (*p* < 0.001) (Table 2).

**Figure 5:**
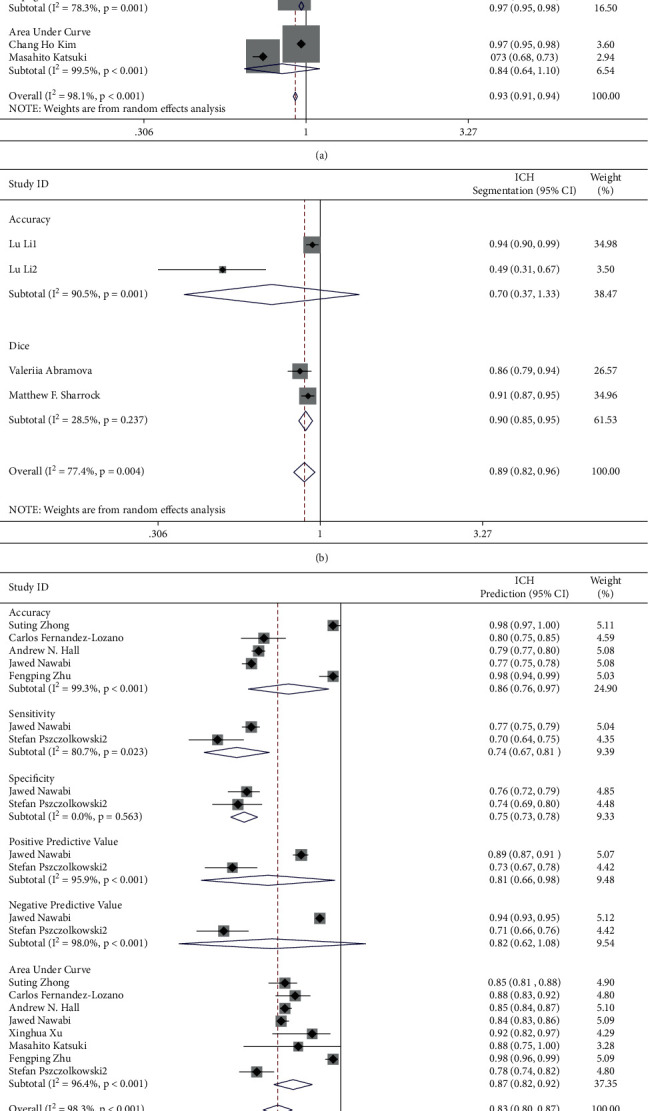
Subgroup analysis of ICH detection/ICH segmentation/ICH prediction/hematoma enlargement.

However, heterogeneity was still high. We considered that different aims of studies might be another source. Therefore, we performed subgroup analysis of ICH detection, ICH segmentation, ICH prediction, and hematoma enlargement (Figure 5). In subgroup analysis of ICH detection, pooled ACC, SEN, SPE, PPV, NPV, and AUC were 0.92 (0.89∼0.95), 0.92 (0.88∼0.95), 0.96 (0.94∼0.98), 0.87 (0.82∼0.92), 0.97 (0.95∼0.98), and 0.84 (0.64∼1.10). Their heterogeneity was 91.6% (*p* < 0.001), 88.2% (*p* < 0.001), 98.0% (*p* < 0.001), 90.3% (*p* < 0.001), 76.3% (*p*=0.001), and 99.5% (*p* < 0.001). In the subgroup analysis of ICH segmentation, pooled ACC and AUC were 0.70 (0.37∼1.33) and 0.90 (0.85∼0.95). Their heterogeneity was 90.5% (*p* < 0.001) and 28.5% (*p*=0.237). In the subgroup analysis of ICH prediction, pooled ACC, SEN, SPE, PPV, NPV, and AUC were 0.86 (0.76∼0.97), 0.74 (0.67∼0.81), 0.75 (0.73∼0.78), 0.81 (0.66∼0.98), 0.82 (0.62∼1.08), and 0.87 (0.82∼0.92). Their heterogeneity was 99.3% (*p* < 0.001), 80.7% (*p*=0.023), 0.0% (*p*=0.563), 95.9% (*p* < 0.001), 98.0% (*p*=0.001), and 96.4% (*p* < 0.001). In the subgroup analysis of Hematoma Enlargement, pooled SEN, SPE, and AUC were 0.73 (0.53∼0.93), 0.70 (0.67∼0.73), and 0.79 (0.73∼0.85). Their heterogeneity was 92.9% (*p* < 0.001), 0.0% (*p*=0.586), and 87.8% (*p* < 0.001).

## 4. Discussion

We performed a novel systemic review and meta-analysis based on studies with high qualities in general. According to total meta-analysis of data, the diagnosis effect of AI was ACC > 0.83, Dices > 0.85, AUC > 0.80, SEN > 0.81, SPE > 0.88, PPV > 0.75, and NPV > 0.91 with a stable outcome of sensibility analysis, which might mean a relatively high agreement and similarity of full-manually diagnostic conclusions, a relatively high authenticity of actual diagnostic conclusions, a relatively low rate of missed diagnosis and misdiagnosis, a relatively high accuracy of screening true ICH patients in people with risk of ICH, and a high accuracy of confirming true no risks of ICH in healthy people. Yet in the subgroup analysis of different aims, in addition to the great mass of outcomes in accord with total pooled effects, there were some invalid outcomes. The AUC of ICH detection was in the range of 0.64 to 1.10, which meant that it might be lack of authenticity for AI to detect ICH. The ACC of ICH segmentation was in the range of 0.37 to 1.33, which meant that the agreement of full-manually diagnostic conclusions might be controversial. For two abovementioned purposes, we considered that the factor-influenced identification of hematoma lesion via AI might be due to the fuzzy boundary between edema and hematoma during absorbing of ICH or in neuroimaging of small hematoma lesion. The NPV of ICH prediction was in the range of 0.62 to 1.08, which meant that AI might not confirm true ICH patients without some outcomes of prognosis. In this solution, we considered that subjectivity, which was unique to humans, might be the mingled influencing factor, because operation of AI was based on the binary system or other algorithmic languages, which was absolutely objective. Classification was usually involved in the assessment of prognosis in clinical work. Hence, when dealing with the common boundary of two grades, AI might not make decisions like humans flexibly, which might be a congenital defect of AI. However, generally, our results resembled the conclusion of meta-analysis published that it was effective for AI to detect brain metastasis [34].

Limits also appeared in our meta-analysis. We only selected articles published in 2021, which might influence the results because we considered that recent AI technologies might remedy previous defects, which would reduce the heterogeneity. Significant heterogeneity was noted in our study like the published meta-analysis of AI used in prevalence and diagnosis of neurological disorders [35], the causes of which might be as follows: (1) the AI models used in these included studies were different. The operation mechanisms or databases of the AI models differed across studies. (2) The research objectives also differed including the detection of ICH, segmentation of ICH in neuroimaging, prediction of prognosis, and hematoma enlargement in ICH patients. (3) ICH patients participated in few studies included not only intraparenchymal hemorrhage but also intraventricular hemorrhage, subdural hemorrhage, or subarachnoid hemorrhage. (4) All the original data used to assess diagnosis effect could be influenced to each other. (5) Number of samples was stark contrast.

In our opinion, although AI as a medical tool will bring great commercial profits to its designers and make the clinical work of doctors more efficient, whether AI systems can be used to diagnose ICH still requires more research evidences with cross-regional, multicenter, and large sample size. The objective and accurate division of hematoma, perihematoma edema, infarction focus, and normal tissue, especially in the stage of hematoma absorption and perihematoma edema developing, is the key for AI to analyze neuroimaging data of ICH. Moreover, when designers and researchers are constructing the database for mechanical learning, some potential problems may appear that the etiology classification of ICH is ambiguous, and the choice of research indicators or dependent variables is not comprehensive enough. Addressing these defects is closely related to continuously optimizing the clinical guideline of ICH. Therefore, while AI is updating, more evidences originated from high-quality and authoritative clinical researches are the real basis of its development of clinical applications.

## 5. Conclusion

Under the background of the fourth industrial revolution, AI might be an effective and efficient tool to assist doctors in the clinical diagnosis of ICH.

## Figures and Tables

**Table 1:** Characters of studies included (“^a^” presented that 2 styles of hematoma volume were studied independently in one study. “^b^” presented that 2 solutions of ICH were studied independently in one study. “^c^” presented that 2 aims were studied independently in one study. “^*∗*^” presented that the same first author performed another study).

Author	Application	AI	ICH patient participation	Conclusion
Ryan A. Rava	ICH detection	Canon's AUTOStroke solution ICH detection algorithm	200	It was able to accurately detect ICH
Chang Ho Kim	ICH detection	A cascaded deep-learning-based automated segmentation algorithm (CDLA)	5702	It can improve diagnostic accuracy in specific doctor groups
Jeremy J. Heit	ICH detection	RAPID ICH (an automated hybrid 2D-3D convolutional neural network application)	308	It is highly accurate in the detection of ICH and in the volumetric quantification
Valeriia Abramova	ICH segmentation	A 3D U-net architecture with squeeze-and-excitation blocks	76	It significantly improved segmentation results
Nico Buls	ICH detection	Aidoc version 1.3, Tel Aviv, Israel	500	It was an adjunct to current real-time radiology workflow
Lu Li1^a^	Big ICH detection and segmentation	U-net-based CNN architectures: convolutional networks for biomedical image segmentation	130	It shows great advantages compared with human experts on hemorrhage lesion diagnosis
Lu Li2	Small ICH detection and segmentation	U-net-based CNN architectures: convolutional networks for biomedical image segmentation	130	It shows great advantages compared with human experts on hemorrhage lesion diagnosis
Matthew F. Sharrock	ICH segmentation	DeepBleed	500	It can be incorporated into the workflow of an ICH clinical trial series
Ruijuan Chen	ICH detection	Restricted Boltzmann machine, deep belief network, stacked autoencoder, and denoising autoencoder	590	It can effectively improve the reconstruction accuracy and prediction speed of the image
Daniel Ginat	ICH detection	Aidoc (Tel Aviv, Israel)	1829	It is associated with a significantly shorter scan view delay
Suting Zhong	ICH prediction	A backbone neural network MF (multifeatures)—dense net	34	The improved method can effectively improve the monitoring performance
Yu Lei1^b^	ICH occurrence detection	A deep ResNet-152 model (CVPR 2016, Las Vegas, NV, USA)	460	It was valuable and could assist in automatic diagnosis of MMD
Yu Lei2	ICH hemorrhage detection	A deep ResNet-152 model (CVPR 2016, Las Vegas, NV, USA)	500	It was valuable and could assist in timely recognition of the risk for rebleeding
Carlos Fernandez-Lozano	ICH prediction	Random forest algorithm	1100	It can be effectively used in long-term outcome prediction of mortality and morbidity of stroke patients
Andrew N. Hall	ICH prediction	Decision tree-based algorithms	284	Patient outcomes are predictable to a high level in patients with ICH
Jawed Nawabi	ICH prediction	Random forest algorithm (python scikit-learn environment v0.20.3)	520	It provided the same discriminatory power as multidimensional clinical scoring systems
Xinghua Xu	ICH prediction	Support vector machine, K-nearest neighbor, logistic regression, decision tree, extreme gradient boosting, random forest	270	Accurate prognostic prediction models of HICH
Masahito Katsuki	ICH prediction	DLframework, prediction one (Sony network communications inc., Tokyo, Japan)	140	The accuracy was superior to previous statistically calculated models
Fengping Zhu	ICH prediction	Support vector machine, random forest	1668	It exhibited good prediction accuracy and efficiency
Qian Chen	HE	Artificial intelligence kit version 3.0.0.R, the least absolute shrinkage and selection operator algorithm	1153	It outperformed the clinical-only model in the prediction of HE
Stefan Pszczolkowski1	HE	Elastic-net parameterizations, selected radiomics-based features using grid optimization	1732	It was better than radiological signs on the prediction of hematoma expansion
Stefan Pszczolkowski2^c^	ICH prediction	Elastic-net parameterizations, selected radiomics-based features using grid optimization	1732	It was better than radiological signs on the prediction of poor functional outcome
Zuhua Song	HE	Naïve bayes (NB), support vector machine, K-nearest neighbor, logistic regression, decision tree, random forest	261	It could improve the discrimination of early HE
Linyang Teng	HE	A model based on convolutional neural network termed U-net	1899	It has higher specificity and sensitivity in the prediction of early hematoma enlargement
Masahito Katsuki^*∗*^	ICH prediction	Prediction one (Sony network communications inc., Tokyo, Japan)	184	It could be performed with high accuracy
Yiqing Zhao	ICH detection	Logistic regression, random forest	890	It performed well for identifying incident stroke and for determining the type of stroke
Joel McLouth	ICH detection	CINAR v1.0 device (Avicenna.ai, La Ciotat, France)	255	It can be effective in the detection of ICH
Yoshiyuki Watanabe	ICH detection	Computer-assisted detection system with U-net	24	It significantly improved the diagnostic performance and reduced the reading time

**Table 2:** Sensibility analysis of overall accuracy/sensitivity/specificity/positive predictive value/negative predictive value/area under curve/dice scores of artificial intelligence used in ICH diagnosis.

Modification	Accuracy (95%CI) (Study)	Sensitivity (95%CI) (Study)	Specificity (95%CI) (Study)	Positive predictive value (95%CI) (Study)	Negative predictive value (95%CI) (Study)	Area under curve (95%CI) (Study)	Dice scores (95%CI) (Study)
The study with the highest quality omitted	*I* ^2^ = 98.7% 0.87 (0.82∼0.92) (Fengping Zhu)	*I* ^2^ = 96.0% 0.85 (0.81∼0.90) (Linyang Teng)	*I* ^2^ = 98.5% 0.91 (0.89∼0.93) (Linyang Teng)	*I* ^2^ = 88.9% 0.88 (0.84∼0.91) (Stefan Pszczolkowski)	*I* ^2^ = 87.8% 0.96 (0.95∼0.97) (Stefan Pszczolkowski)	*I* ^2^ = 97.8% 0.85 (0.80∼0.89) (Linyang Teng)	N/A
The study with the lowest quality omitted	*I* ^2^ = 98.7% 0.88 (0.83∼0.94) (Yoshiyuki Watanabe)	*I* ^2^ = 96.2% 0.86 (0.81∼0.90) (Yoshiyuki Watanabe)	*I* ^2^ = 97.4% 0.88 (0.88∼0.91) (Yoshiyuki Watanabe)	*I* ^2^ = 95.7% 0.78 (0.72∼0.84) (Ryan A. Rava)	*I* ^2^ = 94.8% 0.92 (0.89∼0.94) (Ryan A. Rava)	*I* ^2^ = 98.2% 0.84 (0.79∼0.89) (Zuhua Song)	N/A
Fixed effect model	*I* ^2^ = 98.6% 0.92 (0.92∼0.93)	*I* ^2^ = 95.9% 0.88 (0.87∼0.89)	*I* ^2^ = 98.5% 0.99 (0.99∼0.99)	*I* ^2^ = 95.1% 0.87 (0.86∼0.88)	*I* ^2^ = 94.7% 0.96 (0.96∼0.97)	*I* ^2^ = 98.1% 0.89 (0.89∼0.90)	*I* ^2^ = 28.5% 0.90 (0.87∼0.94)

## Data Availability

All data analyzed during this study are included in this published article.

## Supplementary Materials

Supplementary Materials The PRISMA_2020_checklist of this study.
